# Determinants of financial performance of home-visit nursing agencies in Japan

**DOI:** 10.1186/1472-6963-14-11

**Published:** 2014-01-09

**Authors:** Sakiko Fukui, Kazuhiro Yoshiuchi, Junko Fujita, Sumie Ikezaki

**Affiliations:** 1 Department of Community Health Nursing, Graduate School of Nursing, The Japanese Red Cross University, Tokyo, Japan; 2 Department of Stress Sciences and Psychosomatic Medicine, Graduate School of Medicine, The University of Tokyo, Tokyo, Japan; 3 Department of Community Health Nursing, Graduate School of Nursing, The Chiba University, Chiba, Japan; 4 Graduate School of Nursing, The Japanese Red Cross University, 4-1-3 Hiroo, Shibuya-ku, Tokyo 150-0012, Japan

**Keywords:** Home-visit nursing agency, Financial performance, Profit, Management

## Abstract

**Background:**

Japan has the highest aging population in the world and promotion of home health services is an urgent policy issue. As home-visit nursing plays a major role in home health services, the Japanese government began promotion of this activity in 1994. However, the scale of home-visit nursing agencies has remained small (the average numbers of nursing staff and other staff were 4.2 and 1.7, respectively, in 2011) and financial performance (profitability) is a concern in such small agencies. Additionally, the factors related to profitability in home-visit nursing agencies in Japan have not been examined multilaterally and in detail. Therefore, the purpose of the study was to examine the determinants of financial performance of home-visit nursing agencies.

**Methods:**

We performed a nationwide survey of 2,912 randomly selected home-visit nursing agencies in Japan. Multinomial logistic regression was used to clarify the determinants of profitability of the agency (profitable, stable or unprofitable) based on variables related to management of the agency (operating structure, management by a nurse manager, employment, patient utilization, quality control, regional cooperation, and financial condition).

**Results:**

Among the selected home-visit nursing agencies, responses suitable for analysis were obtained from 1,340 (effective response rate, 46.0%). Multinomial logistic regression analysis showed that both profitability and unprofitability were related to multiple variables in management of the agency when compared to agencies with stable financial performance. These variables included the number of nursing staff/rehabilitation staff/patients, being owned by a hospital, the number of cooperative hospitals, home-death rate among terminal patients, controlling staff objectives by nurse managers, and income going to compensation.

**Conclusions:**

The results suggest that many variables in management of a home-visit nursing agency, including the operating structure of the agency, regional cooperation, staff employment, patient utilization, and quality control of care, have an influence in both profitable and unprofitable agencies. These findings indicate the importance of consideration of management issues in achieving stable financial performance in home-visit nursing agencies in Japan. The findings may also be useful in other countries with growing aging populations.

## Background

Japan demographically became the oldest country in the world in 2004 (19.5% at age 65+) and this rapid aging trend is likely to continue in the future (39.6% at age 65+ by 2050) [1]. The Japanese government has responded to this population shift by promoting home health services for the past two decades, with home-visit nursing positioned as one major service. The government started to promote home-visit nursing as health policy (medical insurance) in 1994 and as social policy (long-term care insurance) in 2000. Since then, there have been many positive revisions of both policies with regard to home-visit nursing [2,3]. However, the number of home-visit nursing agencies has remained at about 6,000 for the last decade (6,047 in 2011) [4]. This is low compared to other community-based services covered by long-term care insurance (e.g. 28,016 home-help services and 28,527 day care services in 2011) [5]. In addition, most home-visit nursing agencies have few staff (the average numbers of nursing staff and other staff per agency were 4.2 and 1.7, respectively in 2011) [6]. This small scale may a major reason for the small number of home-visit nursing agencies with stable financial performance [7-10].

To improve this situation, we have conducted nationwide surveys of home-visit nursing agencies every 4 years [7-10]. We found that an agency’s profitability is influenced by variables associated with management of the agency, such as operating structure, management by a nurse manager, employment, patient utilization, quality control, regional cooperation, and financial condition. However, it is unclear which of these variables has the strongest impact on the agency’s profitability. Thus, in the current study we aimed to examine the determinants of financial performance (agency profitability) among variables related to management (operating structure, management by a nurse manager, employment, patient utilization, quality control, regional cooperation, and financial condition) in home-visit nursing agencies across Japan.

## Methods

### Participants and procedures

The study surveyed 2,912 home-visit nursing agencies, which accounted for 50% of the randomly selected 5,824 agencies listed in the 47 prefectural databases across Japan that publish information about home-visit nursing agencies (as of September 2012). Questionnaires were sent to and collected from these home-visit nursing agencies from November to December 2012.

### Investigation variables

In the questionnaire, the agencies were requested to provide information on financial performance (profitable, stable, unprofitable or unclear) and variables in management of the agency, using the following seven categories: A) Operating structure; B) Management by a nurse manager; C) Employment; D) Patient utilization; E) Quality control; F) Regional cooperation; G) Financial condition during one month (September 2012). These variables (Table 1) were selected based on those found to affect financial performance (profitability) of home-visit nursing agencies in our studies in 2009-2011 [8-10].

**Table 1:** **Relationship between financial performance (profitable**, **stable and unprofitable) and management of home**-**visit nursing agency (in September 2012) (n** = **1204**, **100**%**)**

**Variables**	**Profitable**	**Stable**	**Unprofitable**	
	**n** = **582 (48.3%)**	**n** = **380 (31.6%)**	**n = 242 (20.1%)**	**p value **^**A)**^
***A. Operating structure***	**Average ± Standard deviation or number of subjects (%)**			
Establishment year of a home-visit nursing agency	2000 ± 5.5	2001 ± 5.8	2001 ± 5.7	0.37
Establishment year of a home-visit nursing agency (2011 or later)	28 (4.8)	25 (6.6)	21 (8.7)	0.11
Number of other home-visit nursing agencies managed by the same corporation	7.0 ± 11.3	5.5 ± 8.9	6.6 ± 10.3	0.53
Owned by a hospital	236 (42.0)	133 (37.8)	118 (50.9)	0.007**
***B. Management by a nurse manager***				
Years of experience of nurse manager	7.0 ± 5.0*	6.2 ± 4.7	5.7 ± 4.7	0.001**
Number of home visits per month by the nurse manager	52.0 ± 26.1	49.6 ± 25.0	39.8 ± 21.4 *	<.001***
Experience of nurse manager in management training	421.0 (73.5)	252.0 (67.2)	147.0 (61.8)	0.003**
Nurse manager’s capability with financial management	342 (59.4)	195 (52.3)	104 (44.4)	<.001***
***C. Employment***				
Number of nursing staff (converted to full-time staff)	5.7 ± 3.5*	4.5 ± 2.3	4.1 ± 2.0	<.001***
Number of rehabilitation staff (converted to full-time staff)^a^	1.4 ± 2.9*	0.9 ± 1.9	0.6 ± 1.6	<.001***
Total number of staff (converted to full-time staff)^b^	8.3 ± 5.7*	6.4 ± 4.2	5.3 ± 3.2*	<.001***
Ratio of nursing staff within total staff^c^	74.5 ± 20.6*	78.0 ± 20.4	83.5 ± 18.4*	<.001***
Ratio of rehabilitation staff within total staff^c^	13.0 ± 17.3*	10.5 ± 16.3	7.0 ± 14.1*	<.001***
Ratio of full-time staff^d^	69.8 ± 21.6	71.9 ± 22.7	75.2 ± 22.6	0.007**
***D. Patient utilization***				
Number of patients using long-term care insurance	67.2 ± 49.5*	48.2 ± 35.3	33.8 ± 24.7*	<.001***
Number of patients using medical insurance	23.2 ± 21.1*	15.7 ± 16.2	11.0 ± 10.0*	<.001***
Total number of patients	89.6 ± 61.5*	62.8 ± 43.4	44.5 ± 28.8*	<.001***
Number of patients per nursing staff^e^	18.1 ± 16.8*	14.5 ± 8.8	11.3 ± 7.0*	<.001***
Percentage of patients covered by medical insurance^e^	25.6 ± 14.8	25.1 ± 16.7	26.1 ± 19.2	0.78
Number of new patients of medical insurance	3.4 ± 3.1*	2.4 ± 2.4	1.9 ± 2.1*	<.001***
Number of new patients of long-term care insurance	2.0 ± 2.7*	1.2 ± 1.9	1.4 ± 3.2	<.001***
Percentage of new patients^e^	6.5 ± 4.8	6.4 ± 6.5	7.8 ± 11.4	0.04*
Percentage of bedridden patients^f^	28.8 ± 17.2	30.2 ± 19.3	32.1 ± 21.6	0.09†
Percentage of patients who ended the service due to improvement	4.7 ± 6.1	6.1 ± 8.6	7.6 ± 10.9*	<.001***
Percentage of patients who ended the service due to hospitalized	6.0 ± 8.0	5.8 ± 5.5	7.0 ± 11.9	0.22
Percentage of home deaths among terminal patients^g^	70.3 ± 30.8	67.1 ± 34.8	62.1 ± 35.3	0.04*
***E. Quality control*** (***during the last year***)				
Planned learning opportunities per year for all staff members	524 (90.0)	327 (86.0)	212 (87.6)	0.16
Conducting regularly of patient satisfaction survey	236 (41.3)	127 (34.2)	91 (39.1)	0.11
Conducting regularly of care monitoring among staff	262 (45.9)	139 (37.5)	90 (38.6)	0.02*
Conducting regularly of control of staff objectives by nurse manager	323 (56.6)	136 (36.7)	94 (40.3)	<.001***
Conducting regularly of visits of staff with nurse manager	166 (28.5)	112 (29.5)	67 (27.7)	0.89
***F. Regional cooperation*** (***during the last year***)				
Number of hospitals with cooperation in the community	27.7 ± 23.1*	20.2 ± 17.4	14.7 ± 12.9*	<.001***
Activities for regional contribution: Open lecture for citizens	74 (13.1)	38 (10.2)	18 (7.7)	0.12
Activities for regional contribution: Required to serve as instructors at external lectures	238 (42.2)	100 (26.9)	63 (26.9)	<.001***
Activities for regional contribution: Participation in meetings of local home-care related committees	211 (37.4)	98 (26.3)	61 (26.1)	<.001***
Activities for regional contribution: Received consultations from other health professionals	367 (65.1)	222 (59.7)	129 (55.1)	0.02*
***G. Financial condition***				
Proportion of total income covered by long-term care insurance	64.8 ± 18.0	65.5 ± 18.9	65.3 ± 20.1	0.83
Proportion of total income covered by medical insurance	35.2 ± 18.0	34.5 ± 18.9	34.7 ± 20.1	0.83
Proportion of expenditures going toward compensation	75.2 ± 11.9	73.7 ± 11.2	80.2 ± 9.7*	<.001***
Compensation of full-time nursing staff	4996046.4 ± 2251701.0*	4517809.1 ± 2236688.5	4634608.1 ± 2386036.2	0.02*

^A^When the variable was continuous values, we performed one-way analysis of variance. Furthermore, when significant correlation was confirmed between the financial performance and the variables, we performed Dunnett multiple comparison to compare the “stable” group with “profitable” and “unprofitable” groups. When variables were categorical data, we performed chi-square test. ***P<.001, **P<.01, *P<.05, †P<.10.

^a^:Total number of physiotherapists, occupational therapists and speech therapists.

^b^:Total number of nursing staff, rehabilitation staff and office staff.

^c^:Number of nursing staff or rehabilitation staff converted to full-time staff/number of staff converted to full-time staff.

^d^:Number of full-time staff/number of staff converted to full-time staff.

^e^:Ratio (%) calculated using the total number of patients as the denominator during the 1-month study period (September 2012).

^f^: Ratio of independence in daily life C calculated using the total of J (independence), A (independence in a room), B (independence on the floor) and C (bedridden) as the denominator.

^g^:Number of patients died at home/number of patients who died after receiving terminal care during 6 months from April to September 2012.

### Statistical analysis

First, the relationships between financial performance (profitable group, stable group, and unprofitable group) and each variable in management of the agency from the seven categories (A to G) were analyzed using univariate analyses. The relationships between financial performance and categorical variables were analyzed by χ^2^ test, while one-way analysis of variance was performed to analyze relationships with continuous values. When a significant difference was found between continuous values and financial performance, Dunnett multiple comparison was performed to compare agencies that were “profitable,” “stable,” and “unprofitable”.

Next, to clarify the final determinants related to financial performance, multinominal logistic regression analysis was performed, using all variables in which a significant tendency or difference (p < .10) was found by univariate analysis. In the analysis, we examined the relationship of “profitable” and “unprofitable” agencies, with the “stable” agencies as a reference. After confirming a significant difference in variables of continuous values in the multinominal logistic regression analysis, categorical variables obtained by dividing the continuous value by the average value were used to obtain useful indices for actual financial management. Since the relationship coefficients were high between the total number of staff members and the number of nursing staff, and between the total number of patients and the number of patients covered by medical/long-term care insurance, the number of nursing staff and the number of patients covered by medical/long-term care insurance were examined in multinominal logistic regression analysis, taking multicollinearity into account. All data analyses were performed using SAS statistics software (version 9.2).

### Ethical consideration

The study was performed after obtaining approval from the ethical committee of the Japanese Red Cross University (approval No: 2012–88).

We asked the agencies to return the questionnaire after explaining the objectives and details in a letter requesting participation in the study. In this letter, we also indicated that return of the questionnaire would be considered to indicate agreement to participation.

## Results

### Financial performance (profitability) of home-visit nursing agencies

We sent the questionnaire to 2,993 home-visit nursing agencies, but 81 agencies did not receive the questionnaire due to an unclear address. The final analysis was based on responses collected from 1,340 of the other 2,912 agencies (response rate: 46.0%). Among the 1,340 responding agencies, most showed profitability (582 [43.4%]), followed by 380 that were stable (28.4%), 242 unprofitable (18.1%), 101 unclear (7.5%), and 35 with no answer (2.6%) in September 2012. Therefore, we analyzed a total of 1,204 agencies, excluding those with an unclear answer or no answer regarding profitability.

### Relationship between profitability and management of the agency: results of univariate analysis

Table 1 shows the results of univariate analysis of relationship between profitability and management of the agency. Regarding the operating structure, the number of unprofitable agencies was significantly higher among home-visit nursing agencies owned by a hospital (p = .007).

Regarding management by a nurse manager, profitable agencies had managers with significantly longer experience than stable and unprofitable agencies (p = 0.001). The managers of unprofitable agencies made fewer visits per month compared to those of stable and profitable agencies (p < .001). The managers of the profitable agencies also participated more in management training and had more capability with financial management, compared to those of stable and unprofitable agencies (p = 0.003; p < .001).

Regarding employment, there was a higher number of profitable agencies with a larger number of nursing staff (converted to full-time staff), rehabilitation staff, and total staff (all p < .001). In unprofitable agencies, the ratio of nursing staff to total number of staff members was higher (p < .001) and the ratio of rehabilitation staff was lower (p < .001). The ratio of full-time staff was also higher in the unprofitable agencies (p = .007).

Regarding patient utilization, the number of patients covered by long-term care insurance, the number of patients covered by medical insurance, the total number of patients, and the number of patients per nursing staff were all highest in profitable agencies, followed by stable and then unprofitable agencies (all p < .001). The numbers of new patients covered by medical and long-term care insurance were significantly higher in profitable agencies, compared to the other agencies (p < .001; p < .001, respectively). On the other hand, the proportion of new patients was significantly higher in unprofitable agencies, compared to the other agencies (p = .04). The ratios of bedridden patients and patients who left the agency due to improvement were high or tended to be high in unprofitable agencies (p = .09; p < .001, respectively). The ratio of home deaths among terminal patients during a 6-month period was highest in profitable agencies, followed by stable and unprofitable agencies (p = .04).

Regarding quality control, we found that the ratios of care monitoring among staff and control of staff objectives by nurse managers were higher in profitable agencies than in stable and unprofitable agencies (p = .02; p < .001, respectively).

Regarding regional cooperation, profitable agencies had the most cooperative hospitals, followed by stable and unprofitable agencies (p < .001). Regarding activities for regional contribution, compared to stable and unprofitable agencies, profitable agencies were more likely to have received requests to serve as instructors at external lectures (p < .001), participated in meetings of local home-care related committees (p < .001), and received consultations from other health professionals (p = .02).

Regarding the financial condition, although the proportion towards compensation was high in unprofitable agencies, costs (expenditures) for full-time nursing staff were significantly higher in profitable agencies, compared to the other agencies (p < .001; p = .02, respectively).

### Determinants of financial performance in home-visit nursing agencies: results of multinominal logistic regression analysis

Table 2 shows the results of multi nominal logistic regression analysis of determinants of financial performance in home-visit nursing agencies. Thirteen variables from all seven categories, A to G, were found to be determinants related to profitability, compared with stable agencies (neither profitable nor unprofitable). Profitable agencies were correlated to five variables: 1) hiring of ≥5 nursing staff (odds ratio: 1.65; 95% confidence interval: 1.16-2.34), 2) having ≥70 patients (1.94; 1.33-2.83), 3) care monitoring among staff as an approach to quality control (1.37; 1.03-1.82), 4) control of staff objectives by nurse managers (1.81; 1.36-2.42), and 5) requirement to serve as instructors at external lectures (1.40; 1.02-1.93), compared to stable agencies.

**Table 2:** **Determinants of financial performance in home**-**visit nursing agencies**: **results of multinominal logistic regression analysis**^**A)**^

	**Profitable**	**Unprofitable**
**Variables **^**B)**^	**Odds ratio (95% Confidence Interval)**	
***A. Operating structure***		
Owned by a hospital	1.06 (0.78–1.44)	1.83 (1.25–2.68)**
***B. Management by a nurse manager***		
Years of experience of nurse manager (≥6 years)	1.18 (0.89–1.58)	0.75 (0.52–1.09)
Number of home visits per month by the nurse manager (≥50 times per month)	1.20 (0.90–1.59)	0.45 (0.31–0.66)***
Experience of nurse manager in management training (Having)	0.96 (0.71–1.31)	0.86 (0.59–1.25)
Nurse manager’s capability with financial management (Having)	1.14 (0.85–1.51)	0.79 (0.55–1.14)
***C. Employment***		
Number of nursing staff (converted to full-time staff) (≥5)	1.65 (1.16–2.34)**	0.70 (0.43–1.14)
Number of rehabilitation staff (converted to full-time staff) (≥1)^a^	1.16 (0.62–2.18)	0.34 (0.14–0.84)*
Ratio of nursing staff within total staff (≥75%)^b^	0.85 (0.59–1.21)	1.09 (0.69–1.72)
Ratio of rehabilitation staff within total staff (≥10%)^b^	1.12 (0.63–1.99)	0.25 (0.11–0.58)**
Ratio of full-time staff (≥75%)^c^	0.84 (0.63–1.12)	1.08 (0.75–1.55)
***D. Patient utilization***		
Total number of patients (≥70)	1.94 (1.33–2.83)***	0.52 (0.29–0.92)*
Number of patients per nursing staff (≥70)	1.18 (0.85–1.66)	0.79 (0.51–1.24)
Percentage of new patients (≥7%)^d^	1.31 (0.97–1.77)	1.07 (0.73–1.56)
Percentage of bedridden patients (≥30%)^e^	1.00 (0.76–1.33)	0.84 (0.59–1.20)
Percentage of home deaths among terminal patients (≥65%)^f^	1.11 (0.83–1.49)	0.61 (0.41–0.89)*
***E. Quality control*** (***during the last year***)		
Conducting regularly of care monitoring among staff	1.37 (1.03–1.82)*	1.10 (0.76–1.59)
Conducting regularly of control of staff objectives by nurse manager	1.81 (1.36–2.42)***	1.10 (0.76–1.61)
***F. Regional cooperation*** (***during the last year***)		
Number of hospitals with cooperation in the community (≥20)	1.11 (0.80–1.55)	0.62 (0.39–0.97)*
Activities for regional contribution: Required to serve as instructors at external lectures	1.40 (1.02–1.93)*	1.37 (0.89–2.11)
Activities for regional contribution: Participation in meetings of local home-care related committees	1.27 (0.93–1.74)	1.12 (0.74–1.71)
Activities for regional contribution: Received consultations from other health professionals	0.87 (0.65–1.17)	0.83 (0.58–1.20)
***G. Financial condition***		
Proportion of expenditures going toward compensation (≥75%)	1.11 (0.83–1.48)	3.83 (2.47–5.94)***
Compensation of full-time nursing staff (≥4,500,000 yen per year)	1.01 (0.75–1.37)	1.05 (0.71–1.56)

R2=0.26, Adjusted R2=0.30.

^A^We examined the association of “profitable” and “unprofitable” agencies, with the “stable” agencies as a reference.

^B^Independent variables were used with p values less than 0.10 by the univariate analysis, and numarical variables were divided into two by the average.

***:P<.001, **:P<.01, *:P<.05.

^a^:Total number of physiotherapists, occupational therapists and speech therapists.

^b^:Number of nursing care staff or rehabilitation staff converted to full-time staff/number of staff converted to full-time staff.

^c^:Number of full-time staff/number of staff converted to full-time staff.

^d^:Ratio (%) calculated using the total number of patients as the denominator during the 1-month study period (September 2012).

^e^:Ratio of independence in daily life C calculated using the total of J (independence), A (independence in a room), B (independence on the floor) and C (bedridden) as the denominator.

^f^:Number of patients died at home/number of patients who died after receiving terminal care during 6 months from April to September 2012.

Unprofitable agencies were correlated with eight variables: 1) being owned by a hospital (1.83; 1.25-2.68), 2) visits of <50 times per month to patients’ homes by nurse managers (0.45; 0.31-0.66), 3) hiring of <1 rehabilitation staff (0.34; 0.14-0.84), 4) having <10% rehabilitation staff (0.25; 0.11-0.58), 5) having <70 patients (0.52; 0.29-0.92), 6) <65% home deaths among terminal patients (0.61; 0.41-0.89), 7) having <20 cooperative hospitals (0.62; 0.39-0.97), and 8) having ≥75% income going to compensation (3.83; 2.47-5.94), compared to stable agencies.

## Discussion

In this study, we clarified the relationship between profitability of home-visit nursing agencies and variables related to management of the agency.

First, we found that half of the home-visit nursing agencies were profitable (48.3%), while 31.6% and 20.1% were stable and unprofitable, respectively. Compared to our previous survey on the financial management of home-visit nursing agencies in Japan over the past four years, the number of profitable agencies has remained relatively similar at about 50% (53.5% in 2009, 56.9% in 2010, 52.6% in 2011, 48.4% in 2012), while unprofitable agencies have decreased by about 10% (31.2% in 2009, 29.6% in 2010, 24.9% in 2011, 18.1% in 2012), and stable agencies have increased by 20% from 10% to 30% [7-10]. Therefore, half of the agencies can still not achieve good financial performance, regardless of the recent positive revisions to the insurance cost of home-visit nursing care by the Japanese government. Concrete financial management policies should thus be discussed to support the management of those agencies yet to achieve profitability.

Secondly, we clarified the characteristics of both profitable and unprofitable agencies by comparing them with those of stable agencies. Regarding the scale of each agency, the numbers of nursing staff and patients were both large in profitable agencies, as suggested in previous studies [11,12]. These results show that profitable agencies tend to have more nursing staff and patients. We also found that unprofitable agencies had an average of <1 rehabilitation staff (converted to full-time rehabilitation staff) and that these staff made up <10% of the total staff. Thus, it is possible that profitable agencies have a surplus due to employment of rehabilitation staff. In the latest 2012 revisions to rehabilitation support by home-visit nursing agencies in medical and long-term care insurance, time division of home visits by rehabilitation staff from a home-visit nursing agency was shortened and the cost was reduced [13], suggesting the need for further discussion of the roles and functions of rehabilitation staff at home-visit nursing agencies.

Our results also showed that agencies owned by a hospital and with <20 cooperative hospitals tended to be unprofitable, compared to financially stable agencies. The 2012 revisions included the expanded use of cooperative instruction costs upon hospital discharge by home-visit nursing agencies [14] and the revision of nursing care benefits included additional cooperative instruction costs upon hospital discharge [13]. Such revisions of benefits have been made to medical insurance and long-term care insurance policies. Thus, stable financial management of home-visit nursing agencies could be achieved by cooperating not only with limited organizations in the same corporation, but also with many other local organizations.

Regarding the management system, we found that agencies with managers with <50 patient visits per month were not profitable. Our 2011 study revealed that nurse managers of large-scale agencies had longer working hours and total working time [8]. Taken together, we speculated that nurse managers of large-scale agencies had a larger amount of work due to the increase in the number of visits and due to dealing with daily operations. On the other hand, managers should decrease home visits as much as possible as such work can be done by other staff, and more time should be reserved for management duties, which only managers can complete. Further discussion is thus needed to establish a system that ensures profitability even when the number of visits by managers is decreased.

Regarding patient utilization, poor financial performance was observed in agencies with less than 65% of home deaths among patients who died in the past 6 months. In 2011, we proposed two indices to evaluate the potential of a home-visit nursing agency: the number of home deaths (≥5 per year) and the rate of home deaths (≥50% per year) among terminal patients in a home-visit nursing agency [15]. This study also confirmed the relationship between profitability and these indices, as agencies with a 65% or higher rate of home deaths among terminal patients were considered to have the potential to support patients in the terminal phase until death. Therefore, the present compensation system may be considered appropriate regarding patients in the terminal phase.

Regarding quality control and regional cooperation, we found that agencies which regularly conduct care monitoring among staff and allow control of staff objectives by nurse managers, as well as agencies involved in external lectures for the health-care professionals in the community, would be profitable. The profitable agencies may be more likely to try to implement aggressive quality control because they understand the importance of quality control for nursing care, which directly affects the evaluation of the agency, as shown by previous studies [11,12]. In addition to these internal efforts, profitable agencies may have become well known in their communities and are entrusted to provide regional contributions and to dispatch information to external organizations. It could be interpreted that these agencies can secure a large number of patients, including those with severe conditions such as end-stage diseases, and could attract more nursing staff. This may lead to improved quality of care by nursing staff with greater experience, producing a virtuous cycle that may provide for profitability.

Regarding the financial condition, the relationship between earnings and profitability, which was suggested to be significant in univariate analysis, was eliminated in multivariate analysis. Agencies with 75% or higher of their earnings going to expenditures would not be profitable. According to a survey on home-visit nursing in 1999 by the Ministry of Health, Labour and Welfare of Japan, compensation accounts for 84.9% of expenditures in home-visit nursing agencies [16]. Although these data are somewhat old, this is the last detailed survey and is relevant as the average number of nursing staff in a home-visit nursing agency has not changed much between 1999 (4.4 [total staff: 4.6]) and 2011 (4.6 [total staff: 5.9]) [16]. In the public hospital reform guidelines published in 2007 by the Ministry of Internal Affairs and Communications, the target compensation rate of public hospital staff (for medical income) was determined to be 54.8% in private hospitals and 65.6% in general public hospitals with >50 beds [17]. While the target compensation rate in home-visit nursing agencies differs from that in hospitals because of the difference in scale, it might be more important for the agencies to manage by setting objectives, similarly to the efforts of hospitals. In this study, the objective compensation rate was determined to be 75% to avoid loss in profits in home-visit nursing agencies. This may be used as a financial management target, in addition to the targets of 5 or more nursing staff and 70 or more patients.

The limitations of this study include a low response rate and the inability to demonstrate a causal relationship due to the cross-sectional nature of the study. Agencies with a nurse manager who was not aware of the importance of management and did not make an effort to maintain quality of care in the agency might not have responded to the questionnaire. If the response rate was higher, the determinants of profitability might have been revealed more clearly. In addition, a longitudinal study design would allow establishment of causality between the financial performance and management of home-visit nursing agencies. However, there have been no studies to examine factors related to profitability in home-visit nursing agencies in Japan multilaterally and in detail. Thus, we believe this study will contribute to future development of home health services in Japan and in other countries with a similar rapid aging trend.

## Conclusions

The relationship between financial performance and management was examined in a nationwide survey of 2,912 randomly selected home-visit nursing agencies in Japan from November to December 2012. Data were analyzed from a total of 1,340 agencies. Multinomial logistic regression analyses revealed multiples management variables related to profitable and unprofitable agencies, compared to agencies with stable financial performance. These variables included the number of nursing staff/rehabilitation staff/patients, being owned by a hospital, the number of cooperative hospitals, home-death rate among terminal patients, control of staff objectives by nurse managers, and income going to compensation. Quality assurance of management in home-visit nursing agencies in Japan is important for achievement of a stable financial performance.

## Competing interests

None of the authors has any conflict of interest for the past three years.

## Authors’ contributions

All authors contributed to the conception and design of the study and the acquisition of data. SF analyzed data and all authors contributed to interpretation of data. All authors contributed to drafting the article or revising it critically for important intellectual content, and all gave final approval of the submitted version.

## Pre-publication history

The pre-publication history for this paper can be accessed here:

http://www.biomedcentral.com/1472-6963/14/11/prepub
